# Manual-assisted cognitive therapy for self-harm in personality disorder and substance misuse: a feasibility trial

**DOI:** 10.1192/pb.bp.113.043109

**Published:** 2014-06

**Authors:** Kate M. Davidson, Tom M. Brown, Vairi James, Jamie Kirk, Julie Richardson

**Affiliations:** 1 University of Glasgow; 2 NHS Greater Glasgow and Clyde; 3 NHS Ayrshire and Arran

## Abstract

**Aims and method** To assess the feasibility of conducting a larger, definitive randomised controlled trial of manual-assisted cognitive therapy (MACT), a brief focused therapy to address self-harm and promote engagement in services. We established recruitment, randomisation and assessment of outcome within a sample of these complex patients admitted to a general hospital following self-harm. We assessed symptoms of depressed mood, anxiety and suicidality at baseline and at 3 months’ follow-up.

**Results** Twenty patients were randomised to the trial following an index episode of self-harm, and those allocated to MACT demonstrated improvement in anxiety, depression and suicidal ideation.

**Clinical implications** It is feasible to recruit a sample of these complex patients to a randomised controlled trial of MACT following an index episode of self-harm. There is preliminary support that MACT could be an acceptable and effective intervention in patients with personality disorder and substance misuse.

People with personality disorder and additional substance misuse form a significant population who are hard to treat and have poorer outcomes than those without substance misuse: studies suggest that up to 57% of those with borderline personality disorder have a substance use disorder.^1^ The reasons for poor outcomes are varied and include increased impulsivity and more self-harming behaviour than in individuals with personality disorder alone,^2^ failure to attend follow-up and earlier drop-out from treatment. Repeated self-harm often occurs within a short time of a previous episode, and the importance of deploying interventions early has been stressed.^3^

Manual-assisted cognitive therapy (MACT) is a brief focused therapy for patients with repeated self-harm acts.^4^ It has been used successfully in those with personality disturbance and has been shown to significantly reduce the number of repeated self-harm episodes.^5^ When specifically adapted for a personality disorder population, MACT resulted in a significant decrease in the frequency and severity of self-harm.^6^

We aimed to assess the feasibility of conducting a larger, definitive randomised controlled trial (RCT) of MACT for individuals admitted to a general hospital following an index episode of self-harm who meet criteria for personality disorder with or without substance misuse. Specifically, we aimed to determine whether we could establish recruitment, randomisation and assessment of outcome within a sample of these complex patients. In addition, we aimed to establish whether MACT would be an acceptable intervention and establish a comparison control treatment.

Ethical permission for the study was given by Research Ethics Committee (West of Scotland Committee 5: REC Reference 09/S1001/44).

## Method

### Screening

The study was carried out on one site within the Glasgow Liaison Psychiatry Service. This is a university teaching hospital with a mixed catchment area including some areas of high social deprivation. In the calendar year in which the study was carried out the accident and emergency department saw 61 819 patients of whom 3203 (5.2%) had a psychiatric coding. Of these, 996 (31%) had a self-harm coding. A convenience sample of patients admitted to the medical receiving ward in the hospital after an episode of self-harm was screened. It was not possible to screen consecutive admissions due to staff availability, patient self-discharge before assessment and refusal to be assessed. Patients were screened by age (18-65 years), for the presence of personality disorder and for alcohol and drug misuse. The only exclusion criterion for screening was if participants could not consent.

### Measures

The three tools used for screening were:

1. Standardised Assessment of Personality Abbreviated Scale (SAPAS)^7^ - a brief screening questionnaire that can be used in routine practice to determine the potential presence of a personality disorder
2. Alcohol Use Disorders Identification Test (AUDIT)^8^ - identifying the presence of an alcohol use disorder and providing an assessment of typical daily alcohol consumption
3. Drug Abuse Screening Test (DAST)^9^ - a questionnaire determining the presence of a drug use disorder.

### Further assessment

Patients scoring three or more on SAPAS, with or without substance misuse, were invited to participate. After providing consent, participants were further assessed on the same day as screening or the day after they were discharged from hospital.

The Structured Clinical Interview for DSM-IV Axis II Personality Disorder (SCID-II)^10^ was used to confirm the presence of personality disorder and determine specific diagnoses. The research assistant received SCID-II training and reliability was assessed using an actor. There was total agreement between expert and researcher on personality disorder diagnoses of patients.

Acts of Deliberate Self-Harm Inventory (Acts DSH),^11^ a structured interview, was used to determine the number of self-harm events in the 12 months prior to the index self-harm episode. To determine the extent of current suicidal ideation and psychiatric symptoms, the Beck Suicide Ideation Scale (BSS)^12^ and the Hospital Anxiety and Depression Scale (HADS)^13^ were completed.

### Intervention: MACT or TAU

Manual-assisted cognitive therapy is a brief, 6-session, focused therapy designed to help patients develop a better understanding of their self-harming behaviour and to reduce distress by finding potential ways of resolving problems, including accessing appropriate mental health services. Sessions used a manualised approach and covered topics such as ‘Understanding self-harm’ and ‘What to do in a crisis’. They took place at a location convenient for the participant, usually their local health centre. Treatment as usual (TAU) was referral to a community mental health team and included appointments from a psychiatrist and a community psychiatric nurse. In-patient treatment was given when required. Therapy was delivered to individuals in the community by two therapists, a doctoral-level clinical psychologist and a psychiatrist, both trained and supervised on a weekly basis in MACT by one of the authors of the manual (K.D.).

Patients were randomised to MACT or TAU using a random numbers table with an allocation ratio of 2:1 in favour of MACT. The research assistant, who assessed patients at baseline and outcome, remained masked to treatment allocation throughout the study.

### Follow-up at 3 months post-randomisation

Participants were followed up 3 months after the baseline interview. They completed the BSS, HADS, AUDIT and Acts DSH. The number of psychiatric and nursing appointments, both offered and attended since the index episode, was gathered from an electronic database.

### Statistical analysis

For this pilot RCT analyses, the intention-to-treat principle applied, i.e. analysis based on the initial treatment intent, not on the treatment eventually administered to avoid various misleading artefacts that can arise in intervention research. Differences between the randomised groups for service use, alcohol-related and self-harm outcomes were analysed using non-parametric statistics (Mann-Whitney), whereas *t*-tests were used for measures of suicidal ideation and mood as these data were normally distributed. Statistical analysis was carried out using SPSS version 15 for Windows.

## Results

### Screening

Of the 295 in-patients referred to the hospital liaison psychiatry team during the 7-month screening period, a convenience sample of 82 (28%) was screened. Of the 82 screened, 46 (56%) scored at or above the threshold level on SAPAS (⩾3), which was indicative of personality disorder: 24 (52%) out of the 46 met criteria for substance misuse (5 meeting criteria for both alcohol and drug misuse, 16 for alcohol alone, 3 for drug misuse alone).

### Further assessment

Of those meeting criteria for personality disorder, 26 people (56.5%) were not included in the pilot study. The reasons for this were refusal to consent (*n* = 15), failure to attend baseline interview (*n* = 9), not being a UK resident (*n* = 1) and repeat self-harm, i.e. already in the study (*n* = 1).

Twenty participants were therefore included in the study. Their sample mean score on the SAPAS was 4.55 (s.d. = 1.57, range 3-8). All 20 met diagnostic criteria for at least one DSM-IV personality disorder (as determined by SCID-II). Four people had simple personality disorder and 16 had diffuse personality disorder (personality disorder in more than one cluster).^14^ The most common diagnoses were borderline personality disorder (*n* = 17) followed by avoidant (*n* = 13) and paranoid personality disorder (*n* = 8). There was a significant relationship between the SAPAS score and the total number of personality disorder diagnostic categories met on the SCID-II (Kendall’s tau 0.359, *P* = 0.02).

### Intervention: MACT *v.* TAU

Of those randomised, 11 participants had personality disorder alone (MACT 8, TAU 3) and 9 had both personality disorder and substance misuse (MACT 6, TAU 3). There was no difference between those randomised to MACT and TAU on personality disorder severity (Fisher’s exact *P* = 0.27). Of the 14 participants randomised to MACT, 9 received 4 or more sessions (mean 5.4, s.d = 1.3), 2 never attended, and a further 3 people attended between 1 and 3 sessions.

**Table 1:** MACT and TAU group comparison at baseline and 3-month follow-up interviews

	MACT		TAU		Differences between the groups	
	Baseline *n* = 14	Follow-up *n* = 11	Baseline *n* = 6	Follow-up *n* = 4	Baseline	Follow-up
AUDIT total score, median (IQR)	2.5 (0-23)	3.5 (0-21)	3.5 (0-11.5)	0 (0-19.5)	*U* = 35.5 *z* = –0.54 *P* = 0.61	*U* = 12.5 *z* = –1.1 *P* = 0.31
Typical daily alcohol units, median (IQR)	5 (0-17.5)	2.5 (0-11.25)	3.5 (0-13)	0 (0-21)	*U* = 39 *z* = –0.25 *P* = 0.42	*U* = 13.5 *z* = –0.96 *P* = 0.38
Number of self-harm episodes in previous 12 months, median (IQR)	2 (0.75-3.5)	0 (0-2)	3.5 (0.75-5)	0.5 (0-1)	*U* = 31.5 *z* = –0.883 *P* = 0.39	*U* = 18 *z* = –0.32 *P* = 0.83
Number of non-suicidal self-harm episodes in previous 12 months, median (IQR)	1 (0-9)	0 (0-4.25)	9 (0.75-35.25)	6 (1-23)	*U* = 24 *z* = –1.52 *P* = 0.13	*U* = 5.5 *z* = –1.69 *P* = 0.13
BSS total score, mean (s.d.)	21 (8.41)	12.36 (12.48)	24.33 (5.16)	26 5 (1.92)	*t* = –0.89 d.f. = 18 *P* = 0.38	*t* = –3.64 d.f. = 11.18 *P* = 0.004
HADS total score, mean (s.d.)	28.86 (5.55)	21.73 (9.86)	32.5 (2.59)	33.5 (2.38)	*t* = –1.52 d.f. = 18 *P* = 0.15	*t* = –3.68 d.f. = 12.4 *P* = 0.003

AUDIT, Alcohol Use Disorders Identification Test; BSS, Beck Suicide Ideation Scale; HADS, Hospital Anxiety and Depression Scale; IQR, interquartile range, MACT, manual-assisted cognitive therapy; TAU, treatment as usual.

### Follow-up at 3 months post-randomisation

Fourteen patients were interviewed at follow-up (10 of the 14 in MACT and 4 of the 6 in TAU). One patient (randomised to MACT) died by suicide 2 months after the index episode. This patient attended one of the five sessions of MACT offered.

Those who were allocated to MACT had significantly lower scores on the BSS and on the HADS at 3 months’ follow-up compared with those in TAU, indicating that the MACT group had lower suicidal ideation and were less depressed and anxious (Table 1). Alcohol consumption did not significantly change in either group from baseline to follow-up (MACT: Wilcoxon *Z* = –1.52, non-significant; TAU: *Z* = –0.45, non-significant). No differences in service use in the 3 months after the index admission were noted between the groups.

## Discussion

The aim was to determine whether it would be feasible to recruit a population of individuals with personality disorder, with or without substance misuse, to an RCT of MACT. In addition, we aimed to determine whether or not MACT would be an acceptable psychological treatment within this population following a self-harm act. In addition, we were interested in establishing the TAU for this population and to determine whether we could measure an appropriate treatment effect within this cohort.

As highlighted in the introduction, patients with personality disorder can be difficult to engage. Nevertheless, in this study, we were able to consent 20 patients within a 6-month recruitment window. In addition, 9 out of the 14 randomised to MACT attended four or more sessions. Therefore, in terms of population, we established that it would be feasible to recruit an appropriate cohort to a larger trial of MACT. In addition, we have some preliminary evidence that our MACT intervention was acceptable to this population. Those in MACT who attended between zero and three sessions were consuming more alcohol than those who attended at least four sessions. The numbers who attended 3-month follow-up in the latter group were lower (two out of five) than in the group who attended between four and eight sessions (eight out of nine). Although the numbers are small, it highlights the difficulties of engaging those with harmful drinking patterns and the need to intervene more assertively at the time of the self-harm episode.

In terms of outcome, the group randomised to MACT had significantly lower scores on suicidal ideation and depression and anxiety at 3-month follow-up, indicating that even with brief therapy important reductions in symptomatology are maintained at follow-up. In addition, we were able to establish TAU for this group and map out the typical service contacts for this cohort in the 3 months following an index episode of self-harm.

The study had several limitations. Most importantly, this was a small feasibility study with small numbers and funding for only a short follow-up period, hence the need to be cautious in drawing conclusions. Three months may have been too short a time frame to fully determine engagement in services after a self-harm episode, but an important reduction in symptoms was found with therapy. The sample was, by necessity, a pragmatic sample in that we did not have the resources to screen a consecutive sample. However, we have no reason to believe that our convenience sample is unrepresentative of patients admitted following a self-harm episode. The study suggests that it is possible to recruit, randomise, treat and follow up patients with suicidal behaviour and multiple comorbidities, including substance misuse. A larger, more definitive, study is necessary to determine more fully the clinical outcomes in this group of patients.
